# Particulate bioaerogels for respiratory drug delivery

**DOI:** 10.1016/j.jconrel.2024.04.021

**Published:** 2024-06

**Authors:** Hao-Ying Li, Charalampos Makatsoris, Ben Forbes

**Affiliations:** a Institute of Pharmaceutical Science, King's College London, London SE1 9NH, United Kingdom; b Department of Engineering, Faculty of Natural & Mathematical Sciences, King's College London, WC2R 2LS, United Kingdom

**Keywords:** Bioaerogel particulate platform, Ultralow densities, Improved aerosolisation performance, Amorphous form, Targeted respiratory drug delivery

## Abstract

The bioaerogel microparticles have been recently developed for respiratory drug delivery and attract fast increasing interests. These highly porous microparticles have ultralow density and hence possess much reduced aerodynamic diameter, which favour them with greatly enhanced dispersibility and improved aerosolisation behaviour. The adjustable particle geometric dimensions by varying preparation methods and controlling operation parameters make it possible to fabricate bioaerogel microparticles with accurate sizes for efficient delivery to the targeted regions of respiratory tract (i.e. intranasal and pulmonary). Additionally, the technical process can provide bioaerogel microparticles with the opportunities of accommodating polar, weak polar and non-polar drugs at sufficient amount to satisfy clinical needs, and the adsorbed drugs are primarily in the amorphous form that potentially can facilitate drug dissolution and improve bioavailability. Finally, the nature of biopolymers can further offer additional advantageous characteristics of improved mucoadhesion, sustained drug release and subsequently elongated time for continuous treatment on-site. These fascinating features strongly support bioaerogel microparticles to become a novel platform for effective delivery of a wide range of drugs to the targeted respiratory regions, with increased drug residence time on-site, sustained drug release, constant treatment for local and systemic diseases and anticipated better-quality of therapeutic effects.

## Highlights

• Elucidate the process of fabricating inhalable bioaerogel microparticles (BAMs).
• Summarize methods and interpret mechanisms for gelation of different biopolymers.
• Speculate the drug diffusion and adsorption mechanisms (DDAM) to BAMs.
• Anticipate the major future studies (i.e. DDAM, in-vitro, in-vivo) on BAMs.
• Prospect the comprehensive functions of BAMs and future development.

## Graphical abstract

**Figure:**
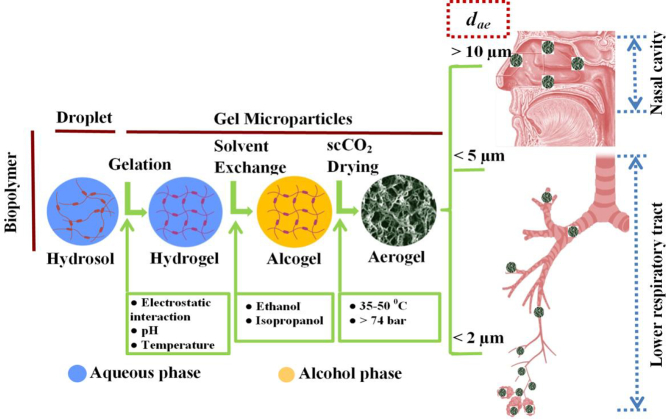

## Nomenclatures

d_ae_: Aerodynamic Diameter (μm)
d_v_: Geometric Diameter (μm)
ρ_b_: Bulk Density (g/cm^3^)
ρ_p_: Powder Density (g/cm^3^)
ρ_s_: Skeleton Density (g/cm^3^)
ρ_t_: Tapped Density (g/cm^3^)
ρ_tp_: True Particle Density (*ρ*_*tp*_ = *ρ*_*t*_/0.794, g/cm^3^)

## 1 Introduction

Dry powders play an increasingly important role for respiratory drug delivery, as they offer a number of distinctive advantages of propellant-free, strong capacity to deliver high dose, avoidance of coordination between inhalation and actuation, and particular suitability for the delivery of biotherapeutics [[1], [2], [3], [4], [5]]. For effective drug delivery to the targeted regions of respiratory tract, there are strict requirements on the *d*_*ae*_ of dry powders. For the IDD, the powders are required to have *d*_*ae*_ > 10 μm [6,7]; While for the DDL, only those powders of *d*_*ae*_ < 5 μm can be inhaled into the lower respiratory tract, and of *d*_*ae*_ < 2 μm can be further conveyed to the alveolar regions [8]. Therefore, it is of great importance to fabricate micronized particles with desired size for effective drug delivery to the targeted respiratory segments particularly the pulmonary regions. However, the micronized particles at submicron have much increased surface energy that can cause them to be inherently cohesive and subsequently severely aggregated, and such situation would detrimentally deteriorate powder dispersibility and aerosolisation performance [[9], [10], [11]]. In order to overcome this big challenge, a number of approaches have been developed, including the use of bigger carriers blended with cohesive micronized drug particles for increasing the powder flowability [[12], [13], [14], [15]], the addition of dispersibility enhancers (e.g. leucine, etc.) into formulations for diminishing the surface energy of microparticles thereafter improving their dispersibility [[16], [17], [18], [19]], and the increase of particle surface roughness by creating wrinkled, crumpled or collapsed morphology for lessening inter-particle contact hence minimizing interparticle cohesion [[20], [21], [22], [23], [24], [25]].

Besides, another important approach to decrease *d*_*ae*_ is through the reduction of *ρ*_*p*_. The behind scientific rationale has been well explained somewhere else [[26], [27], [28]]. Briefly, for unit density sphere, the *d*_*ae*_ can be simply expressed as a function of *ρ*_*p*_ and *d*_*v*_:

(1) $$d_{\mathrm{ae}}=d_{v}\sqrt{\rho_{p}}$$

Clearly, the *d*_*ae*_ is proportional to the *ρ*_*p*_^*1/2*^, so that the reduction of *ρ*_*p*_ can shrink *d*_*ae*_ of dry powders from their geometric dimension of *d*_*v*_ and consequently improve their aerosolisation performance. The reduction of *ρ*_*p*_ can be well achieved by creating porous particles, for which, a number of techniques have been created, including the implementation of heat-sensitive pore-generators (e.g. NH_4_HCO_3_, etc.) into formulations for spray-drying [29,30], double emulsion solvent evaporation technique [31,32], and the PulmoSphere™ technology that has been successfully developed to manufacture porous tobramycin inhalable microparticles with a major deposition in the central and peripheral pulmonary regions, approved as TOBI® Podhaler® for the treatment of chronic lung infections [33,34].

More excitingly, a remarkable technology was invented and can create air-filled dry gel (‘aerogel’) with distinctive characteristics of an astonishingly high PoY (up to 99.8% v/v), ultralow density (as low as 0.003 g/cm^3^ vs. air: 0.001 g/cm^3^), huge SSA (up to 1500 m^2^/g) and large PoV, which therefore award aerogels with unique functions such as supreme thermal/acoustic insulation and high adsorption capacity, leading to wide applications in a range of industrial sectors (e.g. aerospace, building construction, catalysis, et al.) [35]. Unsurprisingly, these innovative features associated with aerogels have also attracted great interests from pharmaceutical scientists, as apparently the large SSA and immense PoV offer a huge space to accommodate drug molecules, and the novel nanostructures may facilitate the creation of new drug delivery systems. Indeed, the large SSA allows sufficient residence sites for drug deposition, which reduces the aggregation of drug molecules and thereafter diminishes the possibility of crystallization, hence instigating the drug state primarily to be amorphous [36,37] and further facilitating drug dissolution and bioavailability improvement. Besides, the base materials can be adjusted in components to create sustained drug release systems [38,39] and the PoS can be tuned by manipulating parameters for synthesis [40], potentially offering a further fine control over drug loading and release profiles. Furthermore, by combining a variety of technologies in component optimization, structure design and surface modification, more complex aerogels can be constructed with hybrid skeletons [41], layer structures [42] and multiple surface functions [43], proposing more potentials for creating novel systems for drug delivery. Furthermore, based on the aforementioned *d*_*ae*_*-ρ*_*p*_ model (Eq. 1), the ultralight characteristic confers aerogels with great capability as a carrier for effective drug delivery to targeted respiratory regions particularly the deep lungs. Indeed, the particulate aerogels were classified as the latest advancement in manufacturing inhalable particles for pulmonary drug delivery [44]. However, this review was primarily aimed to summarize the journey of technology progress on fabricating porous particles for inhalation drug delivery, and the description on aerogel particles was relatively concise and lacked deep analysis.

In this review, we systematically summarize technical progress in this field, with discussion and analysis in depth to produce insights for promoting future development. Firstly, we retrospected the aerogel history, explained the scientific rationale of fabricating aerogels and abridged the technical evolution, in order to understand methodologies accepted in current situation. Then, the advancement in manufacturing particulate bioaerogels for respiratory drug delivery was systematically organized to provide a panorama on this theme. Moreover, a landscape was drawn to demonstrate the roadmap of fabricating drug-loaded BAMs for inhalation, and the key technologies were profoundly discussed and analysed to generate insights for future research directions. Furthermore, the evaluation was made for the combination of aerogel particles with a variety of inhaler devices for effective delivery to targeted respiratory regions. Finally, a perspective was presented to predict the unique features of BAMs and their great potentiality as a novel platform for respiratory delivery of a wide range of active pharmaceutical ingredients.

## 2 Brief history of aerogel

Aerogel was coined by Kistler in 1931, when he originally created a group of novel gels in which the liquid was replaced by gas without interfering with soft skeletons during the processing [45]. To achieve this purpose, Kistler developed an innovative technology called ‘supercritical drying’, where the gel was placed into an autoclave and the temperature / pressure were then increased over the critical points to turn the solvent into a supercritical fluid which was subsequently evacuated to produce a dried gel. At supercritical state, the boundary between liquid and gas phases is eliminated, which consequently eradicates the capillary force and interfacial tension on gel skeletons thus prevents the pore collapse during drying. Eventually, the dried gels can well maintain their solid skeleton structures as identical as those in wet states. The first aerogel was made of silica by sol-gel method that is a powerful tool for tailoring material structures at molecular level and widely employed for constructing multifunctional coatings [[46], [47], [48], [49]]. Kistler hydrolysed sodium silicate (inorganic precursor) to produce silicic acids which were then condensed to form a silica hydrogel, and the hydrogel was subsequently placed into an alcohol (i.e. EA) for long-time solvent exchange to produce the alcogel. By increasing the temperature and pressure over critical points (for EA: T_c_ = 240.8 °C, P_c_ = 6.14 MPa) in a closed autoclave, the alcohol in alcogel turned into a supercritical fluid that was exiled under a controlled manner to form gas-filled aerogel.

Over the past 90 years, the most important progresses in manufacturing aerogels can be abridged as ‘two changes’. The first change was to use organic precursors to replace inorganic ones. At the late of 1960's, the silicon alkoxides (e.g. Si(OCH_3_)_4_, Si(OCH_2_CH_3_)_4_, etc.) were explored as an alternative to inorganic precursors for the preparation of silica sol-gel in alcohol (e.g. CH_3_OH, CH_3_CH_2_OH, etc.). This process can directly produce alcogels, which then eliminated the tedium steps of solvent exchange and made it possible to desiccate the formed alcogels straightforward by supercritical drying [50]. This technical progress massively reduced the time for fabricating aerogels from weeks down to hours. The second change is of utilizing scCO_2_ as a medium to create a new technology named as LTSD, to replace the traditional organic solvent-based HTSD process. The success of this replacement was achieved by Hunt and Tewari (1985) who disclosed the process of utilizing scCO_2_ as a medium for desiccating alcogels to produce aerogels at ambient temperature [51]. The CO_2_ has much lower T_c_ than alcohols, which therefore largely reduces energy consumption during supercritical drying. Moreover, the CO_2_ is much less toxic, non-flammable, non-corrosive and chemically risk-free once leaking. Hence, the scCO_2_-based LTSD technology was thereafter extensively employed to desiccate alcogels for the production of aerogels.

The bioaerogels generally refer to those aerogels in which the solid skeletons are made of bio-based polymers particularly the natural polysaccharides and proteins that have been attracting great interests in biomedical and pharmaceutical fields over years [52]. The reasons for that may come from a firm fact: the naturally-originated biopolymers have exceptional biocompatibility, biodegradability, non-toxicity, non-carcinogenicity, non-immunogenicity, abundance and renewability, and therefore they are claimed as ideal pharmaceutical excipients. These remarkable characteristics, together with the utmost PoY and ultralow densities, would make bioaerogels possess unique features as carriers for drug delivery with unparalleled advantages [53]. The polysaccharides presently are the major natural biopolymers for constructing bioaerogels, among which the alginate, HA, chitosan, cellulose and starch play the key roles [38,39,[54], [55], [56], [57]]. On proteins, the egg white, whey protein, silk fibroin and soy protein have been primarily investigated as the base materials for constructing bioaerogels [36,[58], [59], [60]]. The bioaerogels have been examined as carriers for drug delivery, and they demonstrated the prominent characteristics of adjustable drug loading capacity, controllable drug release profiles, improved drug solubility, augmented bioavailability, broad drug biodistribution and exceptional biocompatibility even at high dose [61,62].

In 2007, the IUPAC eventually made a definition for aerogel, which was a ‘Gel comprised of a microporous solid in which the dispersed phase is a gas’ [63]. Unfortunately, there were insufficiencies with this definition – the pore sizes inappropriately restricted to micropores (< 2 nm) and no ranges defined for PoY. Hence, this concept was stretched to mesopores (2–50 nm) and macropores (> 50 nm) [64] with the PoY generally referred to a range of 80–99.8% *v*/v [65,66]. Presently, the aerogel is designated as a dried porous gel, where the solids are interrelated, nanostructured, homogeneously disseminated and exerted as a skeleton to support the whole structure, with open pores at the size of micro-, meso- and macro- scales and the PoY > 80% *v*/v [67].

## 3 Current situations of bioaerogels for respiratory drug delivery

Although the aerogels have been existed for over 90 years, their use for respiratory drug delivery was only started from this century. At the end of 2001, Lee and Gould (2001) filed a patent named as ‘Aerogel powder therapeutic agents’, opening the application of bioaerogels for inhalation drug delivery [68]. In their disclosure, carbohydrates were mentioned as base materials to make drug-loaded bioaerogels. However, these bioaerogels were actually bulky and had to be micronized by an extra process of jet milling to produce inhalable microparticles, which inherently denatured biologics due to the mandatory high shear force for micronization. This patent was in essence to employ jet milling for the preparation of inhalable BAMs, which is therefore out of the scope of this review.

**Fig. 1:**
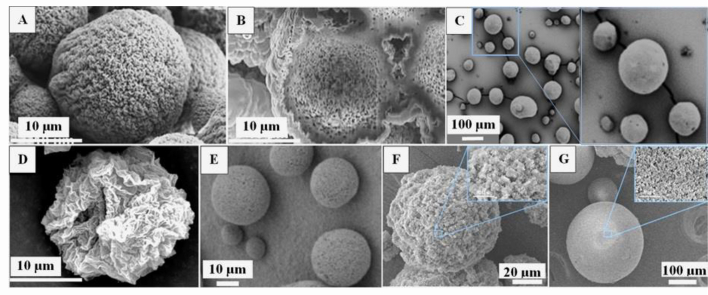
The size and morphology of BAMs fabricated by different technologies. IP: (A) Overall size/surface morphology and (B) Cross section of alginate-based BAMs (bar = 10 μm) [69] (Adapted with permission of Elsevier); EG: (C) Representative alginate-HA BAMs (bar = 100 μm) [70] (Adapted with permission from Elsevier), (D) Representative chitosan-alginate BAMs [72] (Reprinted with permission from MDPI under open-access policy, with modification), (E) Representative cellulose BAMs (bar = 10 μm) [76] (Adapted with permission from American Chemical Society); MD: (F) Egg white BAMs generated by spraying (bar = 20 μm) and (G) Whey protein BAMs fabricated by HG (bar = 100 μm) [75] (Adapted with permission from MDPI under open-access policy).

Until very recently, the BAMs through combined physiochemical approaches were eventually created for effectual respiratory delivery (Table 1). On pulmonary drug delivery, in 2019, the IP technology was explored to fabricate alginate BAMs which displayed open pores across the whole body (Fig. 1A and B), with PoS of 9.0–17.8 nm, PoV of 0.65–1.48 cm^3^/g and SSA of 180–430 m^2^/g. Despite their large *d*_*v*_ of 24 μm, these ultralight microparticles showed a much reduced *d*_*ae*_ of 4.0 μm and can be effectively inhaled into lower respiratory tract [69]. Besides, the EG technology was developed to prepare alginate-HA BAMs that demonstrated PoY of >97% *v*/v, ultralow *ρ*_*t*_ of ~0.03–0.06 g/cm^3^, *d*_*v*_ of 22.5–51.4 μm (Fig. 1C) and *d*_*ae*_ of 3 μm [70], predicting a capability of delivering drugs to central pulmonary regions. Similarly, these BAMs loaded with naproxen demonstrated comparable data with the adjustable *d*_*v*_ (28–179 μm) and *d*_*ae*_ (2.6–33 μm) [71], suggesting a potential for both intranasal and pulmonary drug delivery. Additionally, the EG method was also utilized to prepare alginate-chitosan BAMs that demonstrated a PoS of ~13 nm, PoV of 0.09–0.29 cm^3^/g, SSA of 29.36–86.2 m^2^/g, *ρ*_*b*_ of 0.048–0.19 g/cm^3^, *ρ*_*t*_ of 0.08–1.2 g/cm^3^, *d*_*v*_ of <4.17 μm (Fig. 1D) and *d*_*ae*_ of <2.29 μm, representing an ideal aerodynamic sizes for drug delivery to alveolar regions; And these BAMs possessed adjustable ZP (from −5.98 to +45.3 mV) and potentially can create more applications in drug delivery [72]. These prominent characteristics were confirmed by further studies in which the cisplatin was loaded as a model drug into alginate-chitosan BAMs [73]. On IDD, the MD methods were developed to prepare chitosan-based BAMs for the delivery of clomipramine to nasal cavity [74]. These drug-loaded BAMs had the PoS of 24–26 nm, PoY of 98.6–99.3% *v*/v, PoV of 1.21–1.53 cm^3^/g, SSA to 212–254 m^2^/g, *ρ*_*b*_ of 0.016–0.026 g/cm^3^ and *ρ*_*s*_ of 1.676–2.368 g/cm^3^. They had the *d*_*v*_ and *d*_*ae*_ were 83–317 μm and 13–59 μm respectively, indicating a great potential for IDD. More interestingly, egg white and whey protein were employed as base materials to construct BAMs (Fig. 1F and G) [75]. These protein bioaerogels demonstrated the PoS of 17–34 nm, PoY of 85–96% *v*/v, PoV of 0.4–2.9 cm^3^/g, *ρ*_*b*_ of 0.074–0.239 g/cm^3^, *ρ*_*s*_ of 1.237–1.797 g/cm^3^, *d*_*v*_ of 67–892 μm and *d*_*ae*_ of 24–292 μm, implying a possibility to deliver drugs to nasal cavity. Finally, the cellulose BAMs had a *d*_*v*_ < 20 μm and SSA > 307 m^2^/g, with a big distance observed between each other (Fig. 1E) [76], indicating weak inter-particle cohesion. These fascinating features strongly suggested the cellulose-based BAMs would have enhanced dispersibility and improved aerosolisation performance for efficient DDL.

**Table 1:** Reports on BAMs for respiratory drug delivery.

Year	Biopolymer	Drug Loading (step, loads)	PBAM	PoS/PoV/PoY	ρ_p_	PSD/ZP	SSA	IVIV	Refs
2019 (DDL)	Alginate	Salbutamol sulphate (sol-gel, 3%)	IP G: Ca^2+^ S: EA	PoS: 9.0–17.8 PoV: 0.65–1.48 PoY: 97.7	0.013	*d*_*v*_: ~24 *d*_*ae*_: 2.4 ZP: - 4.4	180–430	IVAP FPF: 49.7; MMAD: 4 IVDR FDC(PBS); RDP: 75 (10*h*)	[69]
2019 (DDL)	Alginate, HA		EG E: RO/S80 G: Ca^2+^; S: EA	PoY: 97–98	*ρ*_*tp*_: 0.0348–0.0634	*d*_*v*_: 22.5–51.4 *d*_*ae*_: 3–10	446–661		[70]
2020 (DDL)	Alginate, Chitosan		EG E: PO/S80, S85 G: ESF; S: EA	PoS: 12.48–13.38 PoV: 0.09–0.29	*ρ*_*b*_: 0.048–0.19 *ρ*_*t*:_ 0.08–1.2	*d*_*v*_: 0.07–4.17 *d*_*ae*_: 0.17–2.29 ZP: −5.98 − +45.3	29.36–86.2	IVS Rats (EII): Verified good biocompatibility of BAMs	[72]
2020 (DDL)	Alginate Chitosan	Cisplatin (scCO_2_, >76%)	EG E: PO/ S80, S85 G: ESF S: EA	PoS: 13.38		*d*_*v*_: 0.433 ZP: +45.3	86	IVS Rats (EII): Reduced drug toxicity after being loaded into BAMs IVDR DB (PBS); RDP: 60 (2 h)	[73]
2021 (DDL)	Alginate, HA	Naproxen (scCO_2_, 19.9–20.6%)	EG E: RO/S80 G: Ca^2+^; S: EA	PoS: 25.4–27.7 PoV: 6.4–7.9 PoY: 97.5–99.8	*ρ*_*t*:_ 0.0069–0.05 *ρ*_*tp*:_ 0.0087–0.0634	*d*_*v*_: 28–179 *d*_*ae*_: 2.6–33	354–759	IVDR DB (PBS); RDP: > 90 (3 h)	[71]
2022 (IDD)	Chitosan	Clomipramin hydrochloride (sol-gel, 35%)	MD: Spray EG E: SO/T80 G: OH^−^ S: IPA	PoS: 24–26 PoV: 1.21–1.53 PoY: 98.6–99.3	*ρ*_*b*_: 0.016–0.026 *ρ*_*s*_: 1.676–2.368	MD *d*_*v*_: 132–317 *d*_*ae*_: 22–59 EG *d*_*v*_: 83–93 *d*_*ae*_: 13–26	212–254	IVS Rats (DPI): Overcome BBB to achieve brain drug delivery	[74]
2022 (IDD)	Egg white Whey protein	Clomipramine hydrochloride (sol-gel, 14.88%)	MD: Spray, HG G: Heat S: IPA	PoS: 17–34 PoV: 0.4–2.9 PoY: 85–96	*ρ*_*b*_: 0.074–0.239 *ρ*_*s*_:1.237–1.797	*d*_*v*_: 67–892 *d*_*ae*_: 24–292	56–346	IVS Rats (DPA): Cross BBB to achieve brain drug delivery	[75]
2020 (PCI)	Cellulose		EG E: P/T80 G: H^+^; S: EA			*d*_*v*_: 5, 20	307, 356		[76]

The single letters of G, S and E refer to the ‘Gelation’, ‘Solvent’ for exchange and ‘Emulsion’ (W/O), respectively.

## 4 Technical process for preparation of inhalable BAMs

**Fig. 2:**
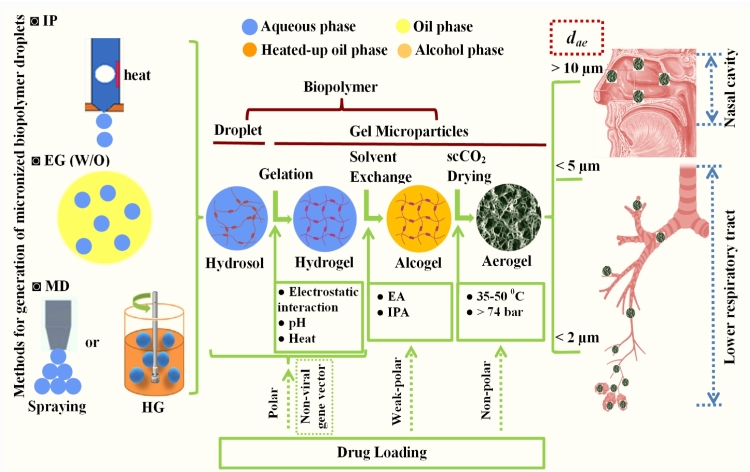
The technical process to prepare drug-loaded BAMs for respiratory drug delivery. This figure was created by an author (H.Y.Li) with the support of BioRender (https://app.biorender.com).

The overall technical process for the preparation of BAMs is shown in Fig. 2. These sequential steps consist of fabricating micronized biopolymer hydrosol droplets, initiating biopolymer gelation, exchanging solvents and desiccating alcogel by scCO_2_ to produce the dried BAMs. These steps are discussed and analysed in details below with insights generated.

### 4.1 Fabrication of micronized biopolymer hydrosol droplets

After polysaccharides or proteins were dissolved in aqueous solutions, the physical dimensions of their molecules would be at nanoscales. Weiss et al. reported the electrophoretic mobility diameters, determined by nano electrospray gas-phase electrophoretic mobility molecular analysis, were 4–14 nm for polysaccharides and proteins with the M.W. of 22 KDa-1.5 MDa [77]. Leclercq et al. stated that the hydrodynamic radii determined by Taylor dispersion analysis were 12–140 nm for polysaccharides with M.W. of 400 KDa-250 MDa [78]. These aqueous colloids of polysaccharides or proteins are also named as hydrosols that are required to be micronized into droplets in the first instance for processing to prepare inhalable BAMs.

Three approaches have been explored to micronize bulky biopolymer hydrosols into aqueous droplets, which are categorized as IP, W/O emulsion and MD (Fig. 2). (1) IP. López-Iglesias et al. creatively employed the thermal IP technology to micronize biopolymer hydrosols into aqueous droplets, where the biopolymer hydrosol was utilized as an ‘ink’ and jetted out by heat to form micronized droplets. In their study, the sodium alginate hydrosol at a defined concentration (0.25–0.4% *w*/w) was poured into the cartridge, settled over an adequate time to release the bubble and then utilized as the ink for printing. Under the air pressure generated by heat, the sodium alginate hydrosol was expelled out through the nozzle as micronized droplets (~ 20 μm) into the CaCl_2_ aqueous bath for immediate gelation to produce HGMs [69]. The IP can generate uniform droplets and thereafter produce monodispersed BAMs; Also, the mild operation facilitates fabricating micronized droplets of biotherapeutics [79,80]. (2) W/O emulsion*.* A W/O emulsion is presently the primary approach to turn bulky biopolymer hydrosols into micronized droplets, which is composed of a dispersed aqueous phase, a continuous oil phase and a phase stabiliser of surfactant. For the preparation of W/O emulsion, the first step is to prepare biopolymer hydrosol. As the polysaccharides and proteins are biomacromolecules, and each of them has its unique chemical structure and specific physiochemical properties. Therefore, it is compulsory to apply individual method for a defined biopolymer to achieve a complete dissolution in water, through selecting appropriate compound form and optimizing dissolution factors (e.g. pH, temperature, time, etc.). Practically, for well dissolving a kind of biopolymer, the sodium salt is selected for alginate [81], the acidic solution is utilized for chitosan [82], and the alkalized solution (i.e. NaOH) is served for cellulose [83], with the adjustment of temperature and time for facilitating dissolution as necessary. The final biopolymer concentrations are usually adapted to the range of 0.25–2.0% *w*/w for assisting the formation of W/O emulsions. The oil phases that were successfully implemented in W/O emulsions consisted of PO [[70], [71], [72], [73],^76^] and SO [74]. The surfactant employed for stabilizing a W/O emulsion was S80 [[70], [71], [72]], S85 [72,73] or T80 [74,76] (Table 1). The mechanical stirring by either marine propeller [70,71] or homogenizer [[72], [73], [74]] was utilized to micronize a bulky aqueous hydrosol into droplets that were homogenously dispersed in a continuous oil phase and stabilized by the surfactant. The W/O emulsion has proved to be an effective approach for micronizing a range of biopolymer hydrosols into droplets, leading to the formation of a variety of BAMs for both IDD and DDL. (3) MD*.* Such mechanical methods as dripping, spraying and HG were employed to directly disperse bulky chitosan or protein hydrosols into micronized droplets [74,75]. Dripping would produce very large bioaerogel particles (*d*_*v*_: up to ~900 μm, *d*_*ae*_: > 200 μm) that were apparently not suitable for inhalation. Spraying can produce microparticles with variable physical dimensions, depended on the types and concentrations of biopolymers and the situations for gelation, of 67 μm (*d*_*v*_) for egg white (15% *w*/w), of 184 μm (*d*_*v*_) for whey protein (20% w/w) and of 132–317 μm (*d*_*v*_) for chitosan (1% *w*/*v*) gelled under varying NaOH concentrations (0.1–1 mol/L). The HG can create microparticles with *d*_*v*_ of ~170–230 μm. The *d*_*ae*_ of BAMs made by spraying and HG was in the range of 22–70 μm, an ideal size for IDD. Clearly, spraying and HG can well disperse bulky biopolymer hydrosols into droplets at appropriate dimensions, leading to the formation of desiccated BAMs with an anticipated *d*_*ae*_ for IDD.

For the methods of W/O emulsion and MD, they are all related to turning bulky biopolymer hydrosols into aqueous droplets through a mechanism of aqueous phase deformation against the P_L_ to achieve break-up for micronization. The P_L_ is defined as P_L_ = γ(1/R_1_ + 1/R_2_), where γ is the interfacial tension, R_1_ and R_2_ are primary radii of curvature. For spherical droplets, the formula can be simplified as P_L_ = 2γ/r where r is the radium of droplet, and clearly the P_L_ would be increased as the reduction of droplet size. Under such situation, it is the laminar flow that generates shear stress to counteract the P_L_ for the deformation and break-up of droplets. This shear stress is quantified as η_c_G, where η_c_ is the viscosity of the continuous (oil) phase and G is the velocity gradient, signifying the greater oil viscosity and larger velocity gradient will increase the shear stress to assist the deformation and break-up of droplets [84,85]. Based on this theory, it may suggest that, for the preparation of bigger BAMs for IDD, the biopolymer hydrosol can be straightforward dispersed into the continuous oil phase under appropriate laminar flow to produce droplets with larger geometric dimensions (dozens to hundreds of microns), leading to the formation of BAMs with greater *d*_*ae*_ to ensure the particle impaction onto the wall of nasal cavity upon inhalation. This hypothesis can be well supported by the studies where the bulky protein hydrosols were directly dispersed as micronized droplets into oil phase under HG, and the produced BAMs had the *d*_*v*_ of 67–317 μm and *d*_*ae*_ of 22–70 μm that were ideal sizes for IDD [75]. This method has distinctive advantage of no added surfactant and thereafter eliminates possible side effects. On the preparation of BAMs for pulmonary drug delivery, the surfactant need to be employed in the W/O dispersion system. The reasons are: (1) to reduce the interfacial tension subsequent diminish the P_L_, facilitating the deformation and break-up of droplets for a fine size reduction (i.e. 1–20 μm); (2) to form a surfactant layer around micronized droplets for inhibiting coalescence and improving stability of dispersed droplets. For W/O emulsion, the surfactants suitable for emulsification have the HLB of 3–8, referring to Spans with HLB values from 1.8 to 8.6. In order to meet the specific requirements on HLB for an individual W/O emulsion, the surfactants can be mixed to finely adjust the HLB through the formula below (Eq. 2).

(2) $${\mathrm{HLB}}_{\mathrm{AB}}={\mathrm{HLB}}_{A}*Α\%+{\mathrm{HLB}}_{B}*Β\%$$

Here, the HLB_AB_, HLB_A_ and HLB_B_ refer to the HLB values of co-surfactants, surfactant A and B respectively, and A% and B% are mass fractions of A and B accordingly [[86], [87], [88]]. Indeed, the majority of reported studies hired S80 and/or S85 to emulsify the hydrosols of biopolymers (i.e. alginate-HA, alginate-chitosan) into continuous oil phases (i.e. RO and PO) and to stabilize the emulsions, into which, an initiator W/O emulsion was added to produce biopolymer HGMs [[70], [71], [72]]. Additionally, the velocity gradient can be attuned to control *d*_*v*_ in the range of 22.5–51.2 μm for alginate and alginate-HA BAMs, referring to their *d*_*ae*_ of 4–11 μm [70]. Further, the optimization of surfactants and the adding sequence of raw W/O emulsions can largely reduce the *d*_*v*_ (< 4.17 μm) and *d*_*ae*_ (< 2.29 μm) of alginate-chitosan BAMs [72]. All these data strongly recommended that the optimization of W/O emulsion systems and the adjustments of operation parameters can control the geometric dimensions of biopolymer hydrosol droplets in W/O emulsions, leading to the formation of dry BAMs with regulated physical and aerodynamic sizes for effective drug delivery to targeted respiratory regions.

### 4.2 Gelation of biopolymer

**Fig. 3:**
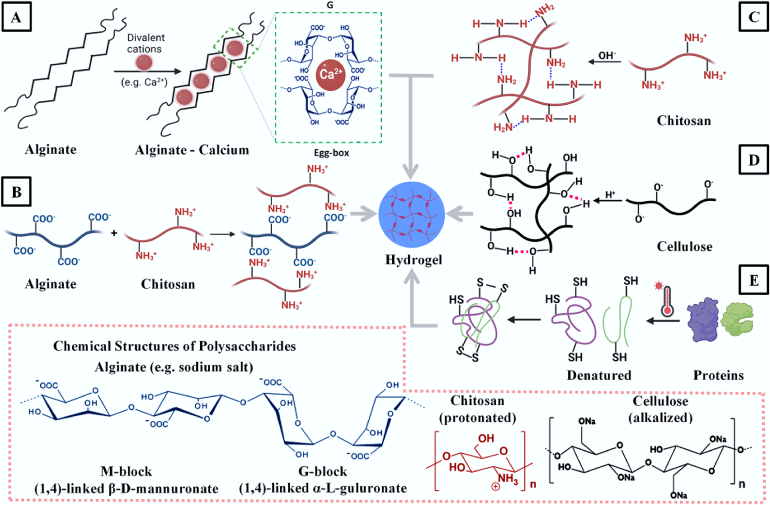
The gelation mechanisms for polysaccharides and proteins. Electrostatic interaction: A) The negative charged alginate chains interacted with positively charged calcium ions to form ‘egg-box’ structure for gelation, B) The negative charged alginate chains interacted with positively charged chitosan chains to form a gel; pH: C) Protonated chitosan neutralized by base and D) Alkalized cellulose neutralized by acid, to be jellified; Heat: E) Thermal-sensitive proteins denatured and jellified by heat. This figure was created by an author (H.Y.Li) with the support of BioRender (https://app.biorender.com).

After the formation of micronized biopolymer droplets, the next step is the initiation of gelation for turning aqueous droplets into HGMs. The method for gelation is individual and entirely depended on the physiochemical properties of the specific biopolymer. These methods are categorized (Fig. 3) and detailed as below.

(1) The use of Ca^2+^. For alginate, its solubility in water is impacted by metal cations, pH and ionic strength. The monovalent metal alginate salts such as sodium alginate are water soluble, while the divalent (i.e. Ca^2+^ in general) alginate salt can only be swollen but insoluble in water, as the divalent metal cations can electrostatically interact with the α-L-guluronic (G) residues (GG) (a component for alginate) to form ‘egg-box’ zig-zag structures which can tightly bind different polymer chains together to initiate gelation [89] (Fig. 3A). According to this principle, the sodium alginate hydrosol is purposively interacted with Ca^2+^ for the generation of hydrogel. López-Iglesias et al. directly printed sodium alginate hydrosol as droplets into CaCl_2_ solution (0.5 M), and the aqueous droplets of alginate hydrosol were immediately gelled to form HGMs [69]. Athamneh et al. dispersed CaCO_3_ particles in sodium alginate W/O emulsion in which the acetic acid W/O emulsion was added. The reaction between CaCO_3_ and acetic acid would release Ca^2+^ that can subsequently jellify alginate to form HGMs [70].
(2) The use of oppositely charged biopolymers. Apart from divalent cations, the positively charged biopolymers (i.e. ionized chitosan) can also electrostatically interact with the negatively charged alginate to induce gelation (Fig. 3B). The process is to separately prepare the alginate and chitosan W/O emulsions which were then mixed together, or to add chitosan and alginate hydrosols into the same oil phase in sequence under vigorous mixing to ensure both hydrosols to be dispersed into micronized droplets. The natural binding driven by electrostatic force will occur between alginate and chitosan chains to form HGMs, through the collision and amalgamation between alginate and chitosan aqueous droplets. Interestingly, the change of chitosan/alginate mass ratios would adjust the ZP of jellified microparticles, suggesting more opportunities to create novel drug delivery systems [72,73].
(3) The use of base or acid. Apart from the mechanism mentioned in (2), chitosan can also be gelled by a base. As a product generated by deacetylation reaction on chitin, chitosan has amino group in the D-glucosamine unit that is repeated along the chain and works as weak base with pK_b_ value of ~6.4 [90]. Therefore, the acid can protonate chitosan for increasing solubility, while the base can turn –NH_3_^+^ back to free –NH_2_ for reducing water solubility, so that the chitosan would subsequently be jellified by chain entanglement and hydrogen bonds (Fig. 3C). It is exactly based on this characteristic to purposively acidify chitosan to prepare hydrosol that is subsequently micronized into droplets by W/O emulsion, and then the aqueous droplets are alkalized to initiate gelation to form chitosan HGMs. Indeed, Menshutina et al. dissolved chitosan into acetic acid solution to form hydrosol. For gelation, this hydrosol was either directly sprayed into NaOH solution or formed into W/O emulsion that was then mixed with NaOH W/O emulsion under HG [74]. While for cellulose, the processes for dissolution and gelation are exactly on the opposite. Cellulose has a linear polymer chain with repeated units of β (1 → 4) linked d-glucose containing the group of –OH that is able to work as an acid to release H^+^, so it can be dissolved in NaOH aqueous solution (6–8% *w*/w) [83]. For gelation, the acid is added to neutralize the solution and reduce cellulose solubility, leading to the formation of cellulose hydrogel (Fig. 3D). Druel et al. utilized NaOH to dissolve cellulose to form an aqueous solution that was subsequently dispersed into oil phase to form a W/O emulsion, into which, the acid (i.e. acetic acid or HCl) W/O emulsion was added to trigger cellulose gelation to produce HGMs [76].
(4) The use of heat. Proteins are thermal sensitive and will be denatured at high temperature, and the deformed protein chains are then entwined and knotted by disulphide bonds to form a hydrogel (Fig. 3E). Based on this characteristic, Lovskaya et al. simply heated up the oil phase (> 80 °C) to jellify proteins (i.e. egg white, whey protein) and turned dispersed protein hydrosol droplets into HGMs [75]. Therefore, for a defined biopolymer, the specific method needs to be employed to achieve completed gelation, and then the HGMs were collected by either centrifuge or filtration for solvent exchange.

### 4.3 Solvent exchange

The solvent exchange is a process of utilizing an organic solvent, which can be miscible with the upstream water and downstream scCO_2_, to entirely replace water phase in the biopolymer hydrogel. The organic solvent generally employed is EA or IPA, and a gradient scheme is implemented where the concentrations of alcohol are continuously increased in a series of co-solvent systems. A typical sequence of alcohol/water ratios is 10:90, 30:70, 50:50, 70:30, 90:10 and 100:0 *v*/v, and the biopolymer HGMs are successively soaked in these co-solvents over sufficient time to entirely remove water residues for a complete transition from hydrogel to alcogel [38].

### 4.4 Supercritical CO_2_ drying

The supercritical fluids can work both as a gas with compressibility and as a liquid with comparable density and solvating ability, and the absence of gas-liquid boundary eliminates the capillary force and interfacial tension that dominate the shrinkage and collapse of gel skeleton during the process of drying. Among all supercritical fluids, scCO_2_ turns out to be an optimized option, as it has the low T_c_ (31.1 °C) and P_c_ (73.8 bar), suggesting a great potential for the treatment of thermal-sensitive substances and a benefit of being a gas at ambient temperature to expel traces of solvents with low energy consumption. Besides, CO_2_ is often a by-product of many other industrial sectors, so that the broad already-existing resources make it become a zero carbon addition for scCO_2_ drying and an economical processing in costs. Additionally, the non-polar CO_2_ in nature has a potent ability to dissolve lipophilic compounds, and its solvating capacity can be enhanced for the dissolution of hydrophilic substance through the combination with polar co-solvents. Further, scCO_2_ is non-flammable, non-corrosive, non-toxic and generally regarded as safe. These unique characteristics make scCO_2_ become the first choice for the applications in pharmaceutics [91,92].

For drying alcogels to produce aerogels, there are two approaches of operating scCO_2_ for desiccation (scCO_2_-D). The first approach (scCO_2_-D1) is more like another time of solvent exchange, where the alcogel is soaked into liquid CO_2_ at low temperature (i.e. 18 °C) over time to allow the alcohol to be exchanged by liquid CO_2_ with repeats as required to produce entirely CO_2_-filled gels (‘carbogels’). Then, the temperature and pressure are increased over critical point to turn CO_2_ into supercritical fluid that is released from carbogels to produce aerogels. This method has been utilized for the preparation of silica aerogels [93]. The second method (scCO_2_-D2) is to implement a condition above the binary critical curve of CO_2_ and alcohol, which will bring CO_2_-alcohol into a supercritical state in which they are entirely miscible. Upon controlled outflow and inflow of scCO_2_ fluid, all traces of alcohol will be removed from alcogels to form aerogels. For drying EA alcogels, in general practice, the CO_2_ liquid from buffer tank (5 °C, 50 bar) is preliminarily pressurized to 85 bar (> P_c_, _CO2_) and warmed up to 35 °C (> T_c_, _CO2_). The scCO_2_ fluid is then conducted from the bottom into an extractor for mixing up with EA inside alcogels, and the increase of temperature and pressure by continuous CO_2_ supply into the extractor will turn CO_2_-EA binary into a supercritical fluid and miscible completely. Subsequently, the supercritical binary fluid is released to expel the extracted EA from alcogel. The continuity of delivering scCO_2_ fluid into and of releasing binary supercritical fluid from the extractor will purge all EA traces out and eventually generate dried aerogel [[94], [95], [96]]. At present, the scCO_2_-D2 is largely employed for desiccating particulate alcogels, and the operation process and parameters for drying alcogels were very similar across all reported studies. Generally, the scCO_2_ (40–50 °C, 80–140 bar) was conducted to flow through (5–200 g/min) the particulate alcogels over a sufficient period (3–6 h) for extracting alcohol residues, followed by depressurization (4–120 bar/h) to produce desiccated BAMs [[69], [70], [71], [72], [73], [74], [75], [76]].

### 4.5 Technical comparisons

The features of formed BAMs, the advantages and disadvantages are listed for further comparisons of IP, EG and MD (Table 2). The IP can produce such BAMs that have the *d*_*v*_ *of* ~ 20 μm with a unimodal and narrow PSD, as all droplets of biopolymer hydrosol are jetted out through the same nozzle for gelation. These BAMs have the *d*_*ae*_ of <5 μm, henceforward they can pass through the upper airway and are primarily deposited into lower respiratory tract, suggesting a great suitability for DDL. The use of EG can potentially adjust the *d*_*v*_ of bioaerogel particles from <100 nm to >100 μm, which makes it promising to create BAMs at the desirable size for targeted drug delivery to the respiratory tract (i.e. nasal cavity or pulmonary regions). In contrast, MD can make HGMs much quicker than IP and EG, but the particles have large sizes with a broad size distribution, from dozens to hundreds of microns. Generally, the MD-made BAMs have the *d*_*v*_ > 70 μm and *d*_*ae*_ > 13 μm, signifying a predominant particle impaction in upper respiratory tract upon inhalation and further suggesting their perfectness as carrier for IDD. Additionally, for EG and MD, it is very convenient and inexpensive to scale up the production of BAMs, which may facilitate their future commercialisation. In terms of the disadvantages, IP requires a specifically designed facility which can print micronized biopolymer droplets directly into a reactant-containing reservoir, where the droplets can be jellified to form HGMs. Besides, due to the high M.W. of polysaccharides, their aqueous solutions can be extremely viscous even at low concentration, so that the continuity of droplet printing is often suffering from the nozzle clogging. Therefore, the optimization of polysaccharide concentrations is highly required to obtain an appropriate ‘ink’ for efficient printing, which may be very tedious and time consuming. Additionally, IP has low productivity in general, and it may be tough and costly to design and build industry-level printers for the scale-up of production. For EG method, the sizes of final BAMs are essentially determined by the dimensions of biopolymer droplets shaped in the initial W/O emulsions; While it may be very challenging to precisely control the W/O emulsions for fabricating biopolymer droplets at the desirable size with narrow size distribution, particularly for the fabrication of fine BAMs for DDL. Besides, after the HGMs are collected from W/O emulsions, they might need additional steps of rinsing by organic solvents for the removal of adsorbed surfactants and oils. For MD methods, they are generally less controllable on the sizes of aqueous biopolymer droplets, and the shaped BAMs hence have large sizes with broad size distribution, which will subsequently make them inappropriate for DDL.

**Table 2:** The technical comparisons of fabricating BAMs.

Technologies	PSD	Advantages	Disadvantages	Refs
IP	● *d*_*v*_*: ~* 20 μm ● Narrow PSD	● Unimodal size distribution ● Suitable for DDL ● No surfactants, no oil phase	● Requirement of specifically designed inkjet printers ● Low productivity ● High risk of nozzle clogging	[69]
EG	● *d*_*v*_: from <100 nm to >100 μm ● Narrow or broad PSD	● Controllable PSD ● Suitable for IDD and DDL ● Easy to scale up	● Potentially challenging for tuning the sizes of biopolymer droplets in W/O emulsions ● Rinsing possibly required for the removal of surfactants and oils from HGMs	[[70], [71], [72], [73]] [76]
MD	● *d*_*v*_: 70–900 μm ● Broad PSD	● Quick process to make protein HGMs ● Suitable for IDD ● No surfactants ● Easy to scale up	● Poor controllability over PSDs ● Too large sizes of BAMs, inappropriate for DDL	[74,75]

## 5 Drug loading, polymorphs and release

**Fig. 4:**
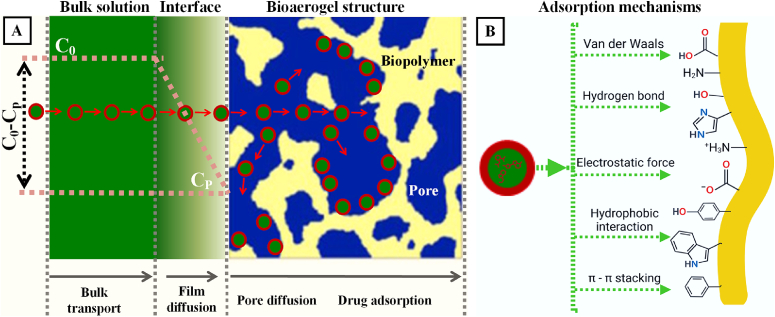
Anticipated processes and mechanisms for drug adsorption to BAMs. A) Drug adsorption processes into BAMs, which is derived from the typical adsorption processes reported somewhere else [104,105], where the microCT bioaerogel structure was adapted from [110] with permission from Elsevier; B) Proposed mechanisms for drug adsorption onto the skeletons of BAMs, derived from [105,107,108]. This figure was created by an author (H.Y.Li) with the support of BioRender (https://app.biorender.com).

As shown in Fig. 2, the drugs can be loaded into bioaerogel in the step of hydro sol-gel, solvent exchange or scCO_2_ drying by post impregnation [97]. In the hydro sol-gel step, the high polar compounds are suitable for being loaded due to their hydrophilic nature, thus, they generally have great water solubility to achieve a high concentration for facilitating drug adsorption to biopolymer chains. Meanwhile, these drugs with high polarity are expected to have poor solubility in alcohol and to be insoluble in non-polar scCO_2_, so that the majority of loaded high-polar drug molecules would be able to be retained in bioaerogel during the processes of solvent exchange and scCO_2_ drying. The solvent for water replacement is generally EA or IPA that has low polarity with the polarity index of 3.9 for IPA and 4.3 for EA, much <10.2 of water [98]. These alcohols are expected to have improved solvating ability over water for dissolving weak polar drugs, leading to the increase of drug concentration and further enhance the drug adsorption onto alcogel. For non-polar drugs, they are certainly to have the greatest solubility in the non-polar scCO_2_ over water and alcohol, and therefore they will be dissolved into scCO_2_ and loaded into the bioaerogel by impregnation in the final step. To achieve this purpose, generally, the drug and BAMs are both placed into chamber, into which scCO_2_ is conducted to dissolve drug and then diffuse drug molecules into bioaerogel pores for adsorption. Indeed, the ionic compound (high polar) salbutamol sulphate has a great solubility in water (0.5046 M; ~170 mg/mL), slightly soluble in EA (0.0054 M, ~1.8 mg/mL) [99] and insoluble in scCO_2_ [100]. Therefore, the salbutamol sulphate was dissolved (0.35% *w*/w) in alginate hydrosol and subsequently adsorbed into the gel skeleton during the gelation. After solvent exchange by EA and scCO_2_ drying, about 3% w/w of drug was retained in desiccated alginate BAMs [69]. This level of drug load is equivalent to 100 μg of salbutamol sulphate per 3.33 mg of dry powders, strongly suggesting a sufficient drug amount for inhalation (100–200 μg / dose) and signifying a great applicability in clinic. Similarly, the highly water-soluble clomipramine hydrochloride was dissolved in aqueous solution (15 mg/mL) and successfully loaded into chitosan and protein bioaerogel particles to achieve a loading efficiency of 35% *w*/w [74] and 15% w/w [75], respectively. These data on clomipramine hydrochloride clearly demonstrated that the BAMs made of either polysaccharide or protein can have feasible capacities for holding sufficient drug amount to satisfy clinical needs (4–5 mg/dose) for intranasal administration [101,102]. While for water-insoluble drugs, the scCO_2_ impregnation was unquestionably selected for drug loading. The lipophilic cisplatin (10 mg) was post mixed with preliminarily-made alginate/chitosan BAMs and placed in a vessel, into which the scCO_2_ fluid (40 °C, 1440 Psi) was introduced over a period (2–4 h). The drug was dissolved into scCO_2_ and impregnated into bioaerogel nanostructures to attain a loading efficiency of >76% *w*/w, indicating that scCO_2_ has a great solvating capacity for lipophilic drugs and a powerful diffusivity into mesopores for drug deposition [73]. This level of drug loads can well meet the clinic requirements (1.5–40 mg/m^2^) on delivering cisplatin to the lungs for the treatment of lung carcinoma [103]. Also, the naproxen was loaded into alginate and alginate-HA BAMs by the scCO_2_ impregnation method. For achieving this purpose, the model drug of naproxen and BAMs were wrapped separately in filter paper and placed at up-down position in an autoclave. The scCO_2_ (50 °C, 200 bar) was then introduced to dissolve naproxen and impregnate drug molecules into BAMs to achieve a loading efficiency of 20.6% w/w and 19.9% w/w for alginate and alginate-HA separately [70]. These studies clearly showed that the drug molecules can be successfully loaded into bioaerogels at a specific step in which the drug can be well dissolved in the solvent for facilitating the adsorption drug molecules into bioaerogel matrices. The drug adsorption is a complex process, including the drug transport in the bulk solution, film diffusion (mass transfer from bulk solution to BAM surface), pore or intraparticle diffusion (drug molecules transported from external surface into interior pores of BAM), and finally the physiochemical reactions (drug molecules adsorbed onto biopolymer active sites) (Fig. 4A) [104,105]. Noticeably, the stagnant liquid film is a barrier for mass transfer, across which, the concentration gradient of C_0_-C_P_ (C_0_, C_P_: drug concentration in bulk solution and on the BAM surface, respectively) is the driving force to propel drug diffusion. The film thickness is a key influencing factor that is related to the viscosity, particle size and liquid interfacial velocity; And the increase of mixing speed would augment liquid superficial velocity, leading to the reduction of liquid film thickness and subsequent enhancement of drug transfer [106]. The adsorption mechanisms are believed to be determined by the physiochemical interaction between drug molecules and biopolymer chains. Considering that biopolymers (i.e. polysaccharides, proteins and derivatives) have plenty of groups of –OH, –NH_2_ and aromatic rings, with potential capabilities of being charged positively or negatively, the drug adsorption mechanisms may fundamentally encompass the van der Waals force, hydrogen bond, electrostatic force, hydrophobic interaction and π–π stacking (Fig. 4B), as suggested from other studies where the pharmaceuticals were removed by adsorption to adsorbents [107,108]. The adsorption kinetic may be related to the chemical structures and physiochemical properties of the biopolymers and drug molecules, drug concentrations, pH, temperature and agitation [109]. Currently, there are no data to disclose details on this important topic of drug adsorption into bioaerogels, and more efforts need to be paid to systematically investigate the actual process, mechanisms, kinetics and control of adsorption of drug molecules into bioaerogels with different base materials, nanostructures and interfacial features, which would provide guidance on loading drugs at a desirable amount for satisfying clinical requests.

In terms of the form of loaded drugs, they were tended to be amorphous after the deposition into bioaerogels. The reason was very likely that the drug molecules were adsorbed as a very thin layer (molecule magnitude) onto the surface due to the massive SSA of bioaerogel, and the limited numbers of drug molecules per unit of surface area were unable to be rearranged into a crystalline lattice. This ratiocination can be well proved by the reported studies. The clomipramine hydrochloride was crystalline originally but turned out to be amorphous after being loaded at hydro sol-gel step into chitosan or protein bioaerogel particles [74,75], and so did ketoprofen lysate [111]. The crystalline ibuprofen loaded into carrageenan bioaerogel particles by scCO_2_ impregnation was transformed to amorphous state [112]. Beyond, this transition was widely observed for a large number of other drugs, consisting of ketoprofen, nimesulide and loratadine loaded into alginate-based bioaerogel particles [113], and of miconazole, terfenadine, dithranol, niclosamide and griseofulvin loaded into silica aerogels [114]. These data clearly revealed that the amorphous form was dominantly associated with drugs loaded into aerogels, which has no relationship to the types of drugs, biopolymer matrices, loading steps, loading methods and operation parameters. In comparison with the crystalline form, the drugs at amorphous state have superfluous thermodynamic possessions of enthalpy, entropy and Gibbs free energy, are situated in a high level of energy and have no need of extra energy to disrupt crystal lattice for dissolution [115]. Therefore, the amorphous drugs are intended to have increased dissolution rates, enhanced solubility and subsequently improved bioavailability, particularly for water-insoluble drugs [116]. More positively, the stability of amorphous state was confirmed to be able to maintain the stability for over 6 months [113]. For inhaled drugs, the increases in drug solubility and dissolution rate play crucial roles for the improvement of their permeation, absorption and bioavailability [117,118]. Therefore, the use of bioaerogel as a new carrier can offer an achievable approach to deliver drugs to the lungs with prodigious potentials for increasing dissolution, augmenting bioavailability and henceforward improving therapeutic effects. Further studies are still needed to investigate the long-term stability of amorphous state for drugs after loaded into BAMs, encompassing the major influencing factors of chemical structures and physiochemical properties of drug molecules, drug-bioaerogel interactions, drug loaded amount and environmental exposures (e.g. humidity, temperature and light, etc.).

On the investigation of drug release from BAMs, the data of IVIV studies were reported. On the evaluation of IVDR from inhalable dry powders, a variety of approaches have been developed by employing a range of devices consisting of DB, FDC, USP apparatus I and II, and the recently established DissolvIt® and RespiCell™; And the buffer systems for drug dissolution include PBS, Gamble's solutions, simulated lung fluid, artificial lysosomal fluid and recently created biorelevant simulated human lung fluid [23,[117], [118], [119], [120], [121], [122], [123], [124]]. Unsurprisingly, these devices and buffer systems were also applied into the studies of investigating drug release from inhalable BAMs. The Franz diffusion cell was utilized to investigate the release of salbutamol sulphate from inkjet-printed alginate BAMs (3 mg, drug load: 3% *w*/w) into PBS (pH 7.4), and the drug was released at a sustained pace for >10 h with about 70% w/w discharged [69]. Also, the DB was employed to evaluate the release of cisplatin in PBS (pH 7.4), where the cisplatin-loaded chitosan-alginate BAMs (5 mg, drug load: 10% w/w) was suspended in a small volume of PBS (i.e. 1 mL) then transferred to a DB that was subsequently tightly sealed and immersed in large volume of PBS (i.e. 100 mL) for drug release. It was observed that the cisplatin can be sustainably released over 2 h with 60% w/w of drug liberated, which was apparently elongated than that (~30 min, 100% w/w released) of pure cisplatin substance. The drug release kinetic was verified as First-order exponential model, suggesting that the rate of cisplatin release from chitosan-alginate BAMs was proportional to the gradient of drug concentration [73]. By using this method, the in-vitro release of naproxen from alginate/alginate-HA BAMs was investigated and demonstrated a sustained drug release for >180 mins, and drug release data were perfectly fitted in Korsmeyer–Peppas model, signifying the drug release rate to be non-Fickian and jointly controlled by drug diffusion and polymer swelling [71]. In IVS, cisplatin-loaded alginate/chitosan BAMs dispersed in saline were delivered to rat lungs by intratracheal instillation. In comparison with the control (i.e. cisplatin only), the use of BAMs as a carrier can reduce the cisplatin-induced lung toxicity, prevent the loss of body weight and considerably lessen the rate of rat mortality [73]. Moreover, the chitosan BAMs loaded with clomipramine were aerosolized for direct delivery to rat nasal cavity, and the peak of drug concentration appeared at 10 mins in blood and 30 mins in frontal cortex and hippocampus. Further, the study on rat depression model confirmed that the intranasal delivery of clomipramine worked effectively to enhance the resistance to chronic stress and improve cognitive functions [74]. These data were confirmed by another study where the protein BAMs were utilized for clomipramine delivery to rat nasal cavity for the treatment of central nervous diseases [75]. Clearly, the clomipramine molecules, delivered by BAMs to nasal cavity, can be well released from biopolymer matrices, penetrate through the nasal mucous membrane, travel through the nerve and avoid the BBB to achieve the target of nasal-to-brain delivery for the treatment of nervous system disorders.

## 6 Evaluation of aerosolisation performance

By regulation, the IVAP of inhalable microparticles is determined by impactors that are based on the inertia of microparticles to achieve impaction under a series of airflow velocities, for fractionalizing microparticles according to their aerodynamic diameters [125]. The in-vitro impaction data can be utilized to predict the in-vivo deposition sites, and the inhalable microparticles with the *d*_*ae*_ > 10 μm, < 5 μm and < 2 μm are primarily deposited in the upper, lower respiratory tract and alveolar regions, respectively. Unfortunately, only López-Iglesias et al. employed the NGI to roughly investigate the aerosolisation behaviour of inkjet-printed alginate BAMs loaded with salbutamol sulphate, and they reported the emitted dose of 97.5% *w*/w, FPF (< 5 μm) of 49.7% w/w and MMAD of 4.0 μm, suggesting a primary deposition of bioaerogel powders in the lower respiratory tract [69]. Apart from this, no other meaningful aerosolisation data were reported for inhalable BAMs, which made it impossible to well compare BAMs with marketed products. Therefore, it is the right time for inhalation scientist to get into this field to systematically investigate the IVIV aerosolisation performance of BAMs, to better understand their potentials for the delivery of both chemical drugs and biologics to targeted respiratory sites for the treatment of local and systemic diseases. Additionally, from current reports, the physical dimensions of BAMs can be controlled by utilizing different fabrication methods, adjusting operation parameters and altering components. The broad size ranges offer great opportunities for preparing different inhaled dosage forms including dry powders [126,127], hydrofluoroalkane suspensions [128,129] and aqueous suspensions [130], with exceptional characteristics that can be expected for highly efficient delivery to targeted respiratory tract (i.e. nasal cavity and pulmonary region). A large range of devices can be potentially employed for aerosolizing inhalable BAMs based on their sizes, loaded drugs and targeted sites, consisting of the traditional DPIs, pMDIs, nebulizers and nasal delivery devices [[131], [132], [133]], also including the recently developed SoftMist™, vibrating meshes and Medspray® inhalers [[134], [135], [136], [137]]. Meanwhile, it is worth noticing that the aerosolisation process can be of energy intensiveness, which may adversely influence BAMs on their features such as sizes, structures, loaded drugs, stabilities and targeted delivery efficiencies. Therefore, it would be scientifically valuable to systematically investigate the variation of physiochemical properties for BAMs upon aerosolisation, in order to improve the formulations, fine tune the fabrication of BAMs, regulate drug loading processes, and optimize BAMs-device combinations for obtaining superlative products. Immense amount of novel and valuable data can be anticipated from these creative studies.

## 7 Expected fates of landed BAMs

**Fig. 5:**
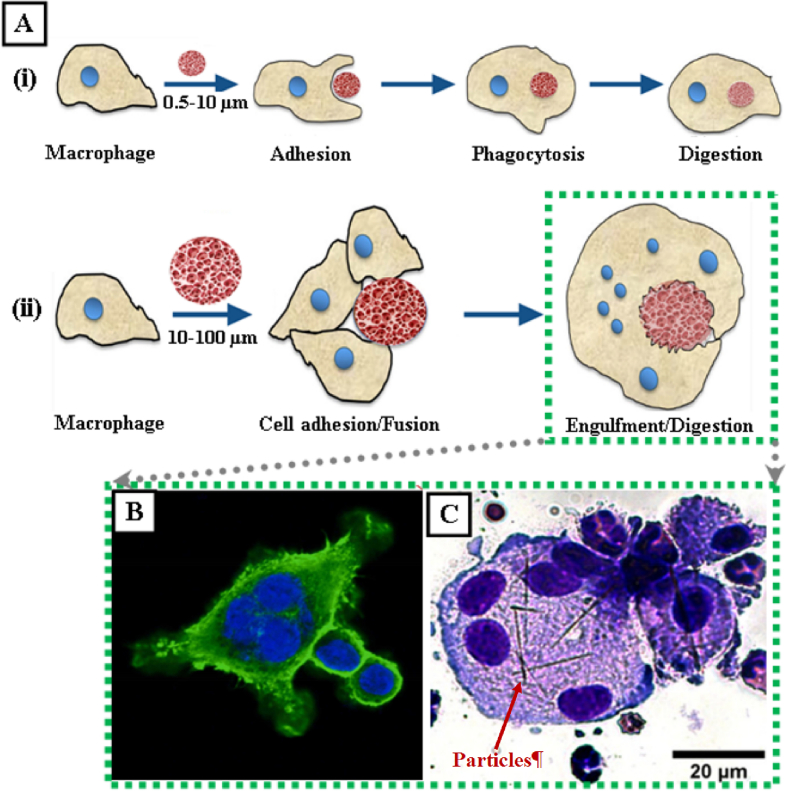
The responses of alveolar macrophage for the elimination of inhaled particles with different sizes. A) The schematic presentation to show the elimination of particles by alveolar macrophages through different processes, including (i) macrophage phagocytosis and intracellular digestion for small particles (*d*_*v*_ = 0.5–10 μm) and (ii) macrophage fusion to form MGCs for the engulfment of big particles (*d*_*v*_ = 10–100 μm) followed by intracellular digestion (Adapted from [146] with the permission from Frontiers under open-access policy); B) and C) Adapted fluorescence and optical microscopy images to show the MGC formed via alveolar macrophage fusion after the lungs exposed to the inhaled particles of cerium dioxide [147] and crocidolite asbestos [148] respectively (Reprinted with permission from Elsevier). This figure was created by an author (H.Y.Li) with the support of BioRender (https://app.biorender.com).

The fates of BAMs in the respiratory tract would be primarily determined by their particle size and the site of deposition. The BAMs of *d*_*ae*_ > 10 μm are expected to be impacted onto the walls of upper respiratory tract, and then they will be either sneezed/spit out or swallowed into gastrointestinal tract for digestion. For BAMs of *d*_*ae*_ between 2 μm and 5 μm, they would be predominantly deposited in the conducting airway. The conducting airway is covered by mucus, inside which the mucins are decisive components for trapping inhaled foreign particles that are subsequently cleared off by mucociliary escalator [138]. Besides, numerous studies have demonstrated strong evidences that various physiochemical interactions can be generated between mucins and biopolymers such as chitosan, cellulose, pullulan and HA [[139], [140], [141], [142], [143]]. Therefore, biopolymer-based BAMs are predicted to be bound to the long and highly-branched glycoprotein chains of mucins, including those that are cell associated, for gaining immobilization on the airway surface. The BAMs would then experience a process of water absorption, swelling, gelation and erosion [144,145], during which, the drug molecules can be dissolved and then sustainably released to generate continuous treatment over an extended period. Finally, BAMs would be collapsed and the degraded fragments can then be expelled out of the conducting airway by mucociliary clearance. For BAMs deposited into the alveolar regions, they have the *d*_*ae*_ < 2 μm but their *d*_*v*_ might be up to dozens of microns due to the ultralow densities. The host defence system in alveolar is the phagocytosis and intracellular digestion by alveolar macrophages. The BAMs with *d*_*v*_ of 0.5–10 μm are favourable targets for alveolar macrophages to recognize and ingest, and then they would be digested intracellularly (Figure 5Ai). While for the BAM with *d*_*v*_ of 10–100 μm, it can not be ingested by a single alveolar macrophage. In such situation, microphage-microphage fusion would occur to form a MGC that has much enlarged body volume for engulfing the big particles (Figure 5Aii) [146]. This mechanism can be evidenced by the studies reported somewhere else, where the alveolar macrophages were observed to experience cell fusion into MGCs for particle engulfment and digestion, after the lungs were exposed to the particles which were either inhaled (i.e. cerium dioxide) (Fig. 5B) [147] or instilled (i.e. crocidolite asbestos) (Fig. 5C) [148] into the pulmonary regions. Noticeably, a duration of 7 days was required for alveolar macrophage fusion to form MGCs for the engulfment of large particles (i.e. ~ 20 μm in length) [148], suggesting a sufficient time potentially available for BAMs to sustainably release drugs for continuous treatment after their deposition in the alveolar regions. Lastly, for the even smaller bioaerogel particles with *d*_*v*_ < 0.5 μm, once deposited in the alveolar regions through the Brownian diffusion, they are intended to be translocated into cells, lymph and blood, followed by a possible distribution around the body, as reported somewhere else [149].

## 8 Perspective over BAMs for respiratory drug delivery

**Fig. 6:**
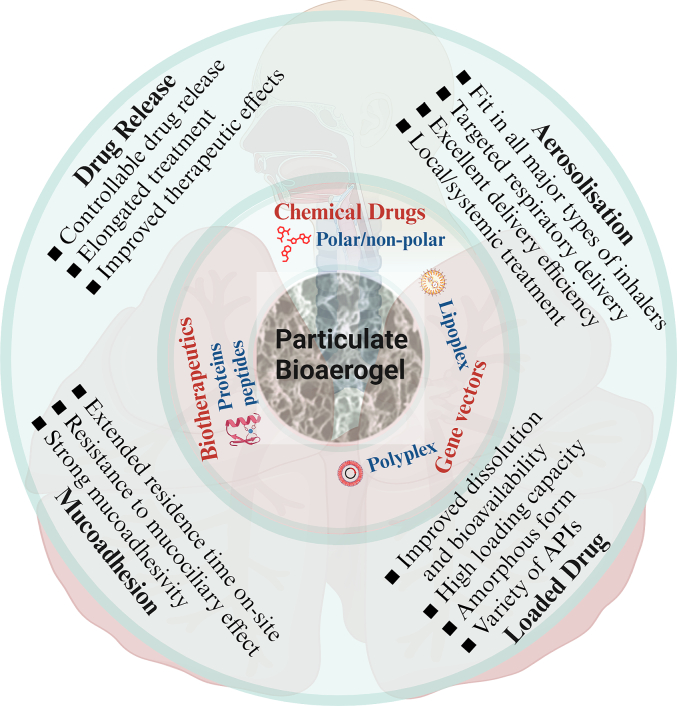
Prospective functions associated with BAMs for respiratory drug delivery. This figure was created by an author (H.Y.Li) with the support of BioRender (https://app.biorender.com).

BAMs possess unique physiochemical properties particularly supreme PoY, ultralow density and therefore much reduced *d*_*ae*_. These BAMs are controllable in physical dimensions, can be sufficiently loaded with polar and non-polar drugs, and are expected to have exceptional aerosolisation performance for efficient drug delivery to the targeted respiratory tracts. Therefore, BAMs are very promising to work as a platform to deliver a range of hydrophilic (e.g. salbutamol sulphate, terbutaline sulphate, etc.) and lipophilic (e.g. budesonide, formoterol, etc.) chemical drugs and biotherapeutics (e.g. proteins and peptides, etc.) to the targeted regions in airway [99,[150], [151], [152]], for the treatment of local (e.g. asthma, COPD, lung cancer etc.) and systemic diseases (e.g. migraine, diabetes, etc.) (Fig. 6). Moreover, the biopolymers particularly polysaccharides generally have strong mucoadhesivity [[153], [154], [155], [156]], counteracting to the mucociliary clearance and subsequently increasing the residence time of drugs on-site, which is one of the critical needs for mucosal drug delivery [157,158]. Finally, the biopolymer aerogels are also able to work as a matrix to control the drug release [159]. We can hence expect that the bioaerogel particles once deposited in the targeted respiratory regions would be able to sustainably release drug molecules to generate continuous local/systemic treatment over an extended period, with the advantages of improved therapeutic effects and of reduced frequencies of drug administration.

Further, the bioaerogel particles are very promising to work as carriers for the respiratory delivery of non-viral gene vectors (Fig. 2). The non-viral gene vectors formed as either polyplexes or lipoplexes [160] can be dispersed into the biopolymer hydrosols, and they would be expected to be adsorbed or encapsulated into the network during the gelation to form HGMs. The vector-loaded microparticles can subsequently be dried either through the normal way of solvent exchange then desiccated by scCO_2_ or directly dehydrated by freeze-drying to get final bioaerogel [54], whichever would be dependent upon the physiochemical properties of non-viral gene vectors. It can be expected that BAMs have a great potential to create comprehensive systems for the respiratory delivery of non-viral gene vectors, with high delivery efficiency to the target regions, improved mucoadhesion on-site for elongation of drug residence time and sustained release of gene vectors for continuous treatment of local and systemic diseases, which therefore intensified the therapeutic effects.

Finally, based on the exceptional capabilities of BAMs for inhalation drug delivery, it can be expected that BAMs would have great potential to be marketed as new inhaled products, delivering a large range of APIs to the targeted regions of respiratory tract for the treatment of local and systemic diseases. For achieving such purposes, the forthcoming work on BAMs will primarily encompass the formulations, the delivery devices, and their combinations to form final products; And these studies would follow a progressive process of formulation development, selections of container closure systems, optimization of delivery systems, in-vitro characterisation, in-vivo studies, and clinical trials. During this process, the desired QTPP for an inhaled BAM product will be established, consisting of the proposed dosage form, the delivery system, strength, purity, stability, and aerodynamic performance. Following QTPP, a list of CQAs will be developed to define the physical, chemical, biological, and microbiological characteristics, which typically include the assay, impurities, net content, moisture content, PSD, DDU, APSD, microbial limits, foreign particle matter and leachables. Besides, other crucial factors, particularly the CMAs and process parameters, also need to be identified and controlled for assuring the desired quality of final product; The CMAs refer to the critical physiochemical properties (e.g. morphic form, impurities, residual solvent content, etc.) of drug substances, excipients, device constituent parts, and packaging materials, and the process parameters represent all key factors influencing the manufacturing (e.g. temperature, humidity, filling weight, and sealing integrity, etc.) [161,162]. These research and development work will be able to facilitate the establishment of standards to control the manufacturing and ensure the quality of inhaled BAM products, which thereafter would accelerate the process of their commercialization.

## 9 Conclusions

A variety of methods have been explored to prepare BAMs that can be controlled in physical dimensions through employing different preparation methods and altering operation parameters. Based on their supreme PoY and ultralow densities, the bioaerogel particles have massively shrunk *d*_*ae*_, provide a great capacity to accommodate polar/non-polar drugs at sufficient amount for satisfying clinical needs, facilitate the formation of drug amorphous state and can construct a system for controlled drug release. These exceptional features strongly suggested the bioaerogel particles can work as a platform to carry chemical drugs, biologics and therapeutically active nanoparticles for the efficient delivery to the targeted respiratory regions. The deposited bioaerogel powders potentially can extend drug residence time, control the drug release for continuing healing action and improve the therapeutic effects for the treatment of local and systemic diseases. It is highly promising that these unique bioaerogel particles can grow up into the next generation of carrier for successful drug delivery to the targeted regions of respiratory tract, with much improved overall therapeutic effects that can be anticipated.

## CRediT authorship contribution statement

**Hao-Ying Li:** Writing – review & editing, Writing – original draft, Investigation, Formal analysis, Data curation, Conceptualization. **Charalampos Makatsoris:** Funding acquisition. **Ben Forbes:** Writing – review & editing, Resources, Funding acquisition.

## Data Availability

Data will be made available on request.
